# Crime and Its Punishment in the United States

**Published:** 1852-10-01

**Authors:** 


### CRIME AND ITS PUNISHMENT IN THE UNITED STATES.

The Inspector of Prisons reports the following ratio of crime to population:—"New York, 1 in 1608; Massachusetts, 1 in 2232; Connecticut, 1 in 1700; Maine, 1 in 5374; New Hampshire, 1 in 4376; Virginia, 1 in 6856; Kentucky, 1 in 7238; Maryland, 1 in 1336; Pennsylvania, 1 in 4022; New Jersey, 1 in 2010." Crime is more severely punished in Virginia than in any of the other States enumerated. The reformation of the offender can only be effected by the enlightenment and culture of his religious, moral, and intellectual faculties, and by preserving and improving his physical powers. An inspection of State prisons will satisfy any one that these indications are not fulfilled by long sentences. Most men who have been confined for long terms are distinguished by a stupor of both the moral and intellectual faculties; they become mere machines; long disused to the exercise of their own volitions, and subjected to an unvarying routine of occupations and of objects, the noblest powers of their natures fall into decay, while the mere instinctive and animal faculties are those which remain in exercise. Even hope dies within them; and not unfrequently insanity in its most frightful forms completes the wreck of all their faculties. Reformation is then out of the question, and the power of providing for their own livelihood is for ever destroyed. Those who are most familiar with the history of criminals know that pecuniary necessities are the chief springs of crime. Even those who enter the path of criminality through the portals of the grog-shop, the brothel, or the gambling-house, are constrained to adopt this course, because those agencies have deprived them of all other means of providing for their wants. Prisoners are punished in the following extraordinary manner:—"The form of the machine is that of the common stocks, with a reservoir of water above it, having a head of fifty-four inches, measuring from the surface of the water to the perforated plate at the end of the discharging tube. The offender, being stripped of his clothing, is placed in a sitting posture in the stocks, with his feet and hands securely fastened, and his head contained in a sort of hopper, the bottom of which encircles his neck so closely that the water will not run off as fast as it can be let on, the water being under the control of the keeper by means of a cord attached to a valve in the bottom of the reservoir. From the perforated plate the water falls about eighteen inches, when it strikes the head of the convict immovably fixed, thence passing over the whole surface of the body. When the reservoir is full, the force of the blow upon the head is nearly equal to a column of water seventy-two inches in height. This force is somewhat reduced by the intervention of the perforated plate, a late modification in the instrument. To the mechanic who calculates the influence of mere matter upon matter, the power of this column of water must possess considerable importance. But to the physiologist, who can alone judge with any degree of correctness of the influence of a stream (generally at 32 degrees Fahrenheit) falling upon the head, and thence covering the whole body, the suffering induced and danger incurred must appear momentous in the extreme. The kind of punishment next in frequency inflicted in this prison (Auburn) is yoking. The yoke is formed of a flat bar of iron four or five inches wide, and from five to six feet in length, with a moveable staple in the centre to encircle the neck, and a small one at each end to surround the wrists. All these staples are so arranged, that by turning screws on their protruding ends, on the back of the iron bar, they can be tightened to any degree deemed expedient. The weight of the lightest yoke is thirty-four pounds avoirdupois; and some of them, I believe, weigh forty. The principal objection to this punishment is, that the yoke bears too heavily on the cervical vertebræ. Most persons are aware of the unpleasant, and, in fact, insupportable, sensation produced even by the weight of the unbuttoned coat and vest pressing upon the back of the neck. Under the weight of this instrument the convict cannot retain the erect posture even for a few minutes consecutively, but is forced to bend forward in his continual writhings, which brings the entire weight of the bar upon the lower cervical vertebræ. The arms are generally stretched to their full length, and from steady tension of the nerves are benumbed, while the hands turn purple, and at times become much swollen. In several instances I have placed my fingers beneath the yoke, and found the pressure so great that it was actually painful to me."

